# Supplementary material for: Can a Two-Dose Influenza Vaccine Regimen Better Protect Older Adults? An Agent-Based Modeling Study
Source: Vaccines (Basel). 2022 Oct 26;10(11):1799. doi: 10.3390/vaccines10111799 (PMC9697266; doi:10.3390/vaccines10111799)
Supplement: Supplementary file 1 [file vaccines-10-01799-s001.zip › vaccines-1979239-supplementary.pdf]

Supplementary Material

Table of Contents

1. Framework for Reproducing Epidemiological Dynamics (FRED) Details
2. Model Inputs
    - Table S1. Model inputs
3. Influenza Model
    - Figure S1. Influenza model schematic
4. Vaccination Model
    - Figure S2. Influenza vaccination schematic
    - Figure S3. Representative agent relative vaccine effectiveness
5. Simulation Scenarios
    - Table S2. Simulation scenarios
6. Supplemental Results
    - Table S3. Percentage reductions in cases and hospitalizations compared to base model of one influenza vaccine dose
7. References

1. Framework for Reproducing Epidemiological Dynamics (FRED) Details

FRED is an agent-based (agent defined as an individual person) modeling platform that uses census-based synthetic populations with household demographics, incomes, and locations statistically realistic at the US census block group level. FRED agents spread infectious conditions through agent interactions in modeled locations (schools, workplaces, neighborhoods, and households). FRED models are composed of conditions (e.g., influenza) that include disease definitions as a series of health states that individuals can progress between. For example, an individual within the influenza condition can transition to other states within that condition (e.g., those in the susceptible state may transition to the exposed state) based on probabilities and time periods for evaluation of state changes. FRED has been used to model influenza and other diseases and conditions and has been described in detail.[1-3]

2. Model Inputs

FRED was used to track a population of 1.2 million people created from the 2010 Allegheny County Pennsylvania census. This population resided in urban or suburban areas, and infection was allowed to spread in schools, workplaces, neighborhoods, and households.

A single and two dose vaccination strategy was modeled based on the timing of the influenza season. Input parameters and simulation description are included in Table S1. A one-season flu model with simulation starting August 15 and ending May 31 was used. The influenza season started in the simulations by inserting 50 cases on a range of seeding dates, with an effective reproductive rate of 1.5, resulting in peak cases from December to March (Epidemiologic curve by seeding date, Figure 1).

Table S1. Model Inputs

Inputs	
Population	1,218,695 agents derived from Allegheny County census population
Influenza Model States	
E exposed	Duration: number of days drawn from a lognormal distribution with μ = 1.9 and σ = 1.23
Ps pre-symptomatic	1 day
Is symptomatic infectious	Duration: number of days drawn from a lognormal distribution with μ = 4 and σ = 1.5. 75% of age<65 become symptomatic, 100% of age ≥65 become symptomatic
Ia asymptomatic infectious	Duration: number of days drawn from a lognormal distribution with μ = 5 and σ = 1.5
Hospitalization (probability applied to Symptomatic infectious agents)	Rates by age group: age 0-4, 6.97%; age 5-17, 2.74%; age 18-49, 5.61%; age 50-64, 1.06%; age 65-74, 6.0%; age 75-84, 12.0%; age 85+, 30.0%
Death (probability applied to Hospitalized agents)	Rates by age group: age 0-4, 0.80%; age 5-17, 0.80%; age 18-49, 3.10%; age 50-64, 5.75%; age ≥65, 7.73%
Prior Immunity applied at simulation start	Rates by age group: age 0-4, 20.9%; age 5-17, 15.4%; age 18-49, 11.1%; age 50-64, 13.4%; age ≥65, 3.6%,
Susceptibility to infection	By age group: age 0-74, 1.0; age 75-84, 1.5; age 85+, 2.5
Vaccine Uptake	Rates by age group: age 0.5-17, 63.8%; age 18-49, 38.4%; age 50-64 50.6%; age ≥65, 69.8%<br>100% uptake for second dose for all who receive first dose<br>70% uptake for second dose for all who receive first dose
Vaccine Efficacy	40% for first dose

	40% and 30% for second dose
Waning of Vaccine Efficacy	7% for age <65<br>7% and 10% for age ≥65
Simulation Parameters	
Simulation Period	August 15 to May 31
Simulations Per Scenario	100

3. Influenza Model

This study used a modified Susceptible-Exposed-Infectious-Recovered (SEIR) model (Figure A1)[1] with the addition of varying susceptibility to infection by age. Susceptibility varied by prior immunity, age, and vaccine waning. To capture immunity from prior year infections on simulation start, agents in the model were initialized with immunity to influenza at rates based on CDC reported influenza infections for the 2019-20 season (Table S1) [4] as previously described.[1] Prior infection reduced susceptibility to influenza infection by half. Older agents (age 75 and up) were assigned higher susceptibility to influenza infection, in comparison to younger age groups, of 1.5 times the base susceptibility for age 75-84 years and 2.5 times for age 85 years and older in order to generate case numbers in these population subgroups to produce estimated annual cases per 100,000.[5] Vaccination reduced susceptibility as described below. For those under age 65, agents moved either to an infectious, symptomatic infectious (Is) state with a probability of 75% or an asymptomatic infectious (Ia) state with a probability 25% (Figure S1). Agents in the Ia state were half as infectious as agents in the Is state. All individuals age ≥65 were symptomatic (Is). This model included a Pre-symptomatic state lasting 24 hours for symptomatic individuals. Susceptibility was reduced to 0 after infection.

Only symptomatic infectious agents may be hospitalized; only agents in the hospitalized state can die. Hospitalization estimates for symptomatic illness were set to best achieve reported

national CDC estimates of 9% hospitalization rates in ages 65 and up [5] with the highest hospitalization rates in the age range strata of 75-84 years and 85 years and up [6,7] in the Allegheny County population. Hospitalization estimates for cases of symptomatic illness were 6% for age 65-74 years, 12% for age 75-84 years, and 30% for age 85 years and up, representing a 2- and 5-fold for increase for the oldest age strata respectively, compared to age 65-74.[6,7]

Figure S1. Influenza Model Schematic. Components of SEIR model are shown in dark gray with yellow representing infected symptomatic. Reported outcomes were infected symptomatic (yellow) and hospitalizations (blue).

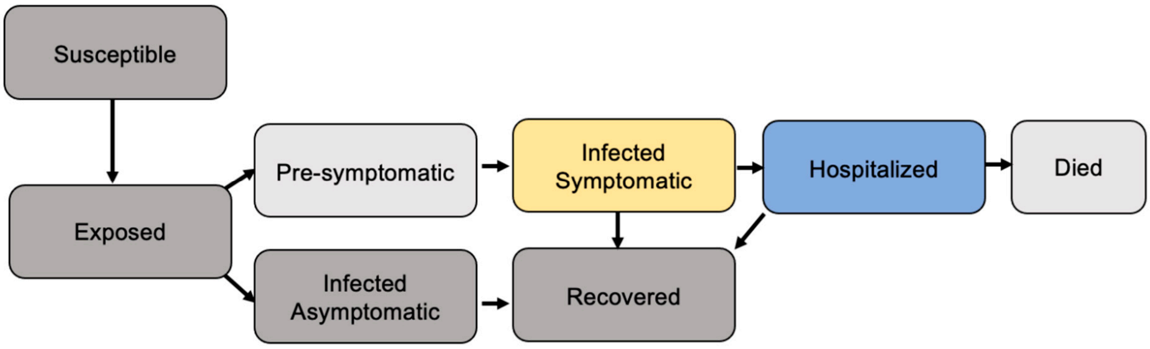

4. Vaccination Model

For flu vaccination, all age groups were vaccinated as per CDC 2019 reporting, including 68.7% of agents ≥65 (Table S1).[8] Vaccine effectiveness (VE) was 40% for all for both doses based on reported VE in 2019 of 39% and a mean of 40% from a reported range of VE across years (2004-05 through 2019-20 seasons) of 10% to 60%.[9] Waning VE was 7% for age <65 years and 10% per month for age 65 years and up.[10,11] Modeling steps are described in Figure A2 and a representation of VE as modeled is shown in Figure A3. Vaccination caused a decrease in susceptibility to influenza of 40% in all agents. Vaccine immunity was effective 14 days after vaccination in the model and was maintained for 14 days before daily waning began (Figure

S2).[10] Vaccination induced immunity waned at a rate of 7% per month for all agents under 65 and at a rate of 10% per month for agents ≥65 over the simulation (Figure S3).

Figure S2. Influenza Vaccination Schematic. For all models, the same schematic was used for agents <65 years. For agents ≥65 in the single dose model, Older Immune Wane is the final state. For agents ≥65 in the two-dose model, Second Immune Wane is the final state. Vaccinations are shown in blue, vaccine immunity in green, and waning immunity in yellow. Additional details are in Table S1.

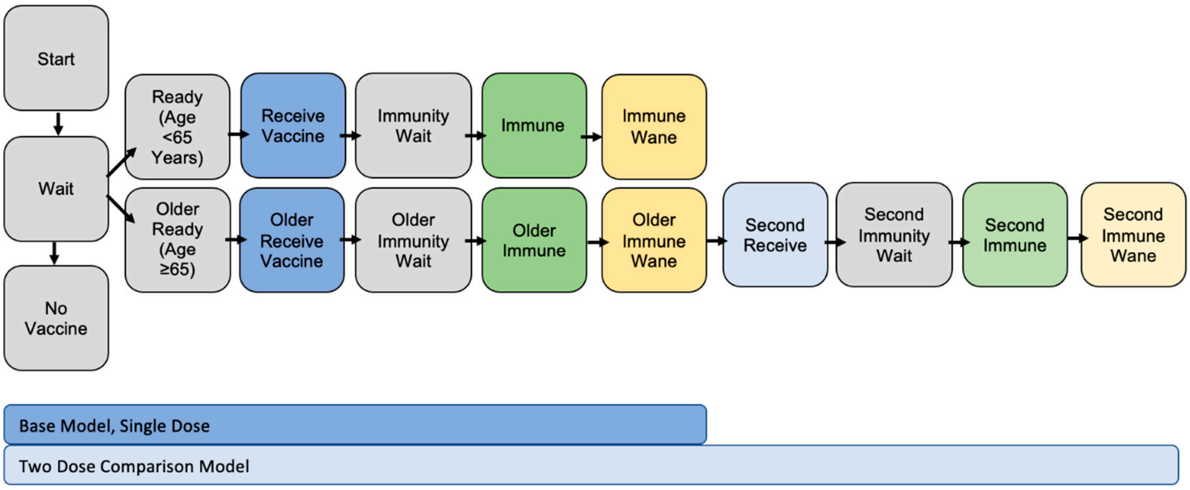

Figure S3. Representative agent relative vaccine effectiveness (VE) with daily waning at 10% per month with a single influenza vaccine dose (solid line) and with a second influenza vaccine dose 90 days (arrow) after the first (dotted line). Data were modeled with full immunity attained at two weeks after vaccination and sustained for two weeks after vaccination. Following dose 2, waning ceased during a two-week waiting period prior to return to full VE of 40%. The lowest VE attained with two doses and 10% waning was 17% (light blue horizontal line). The table below shows potential vaccination months by timing of dose 1 (darker blue), dose 2 (lighter blue), and periods of waning (yellow).

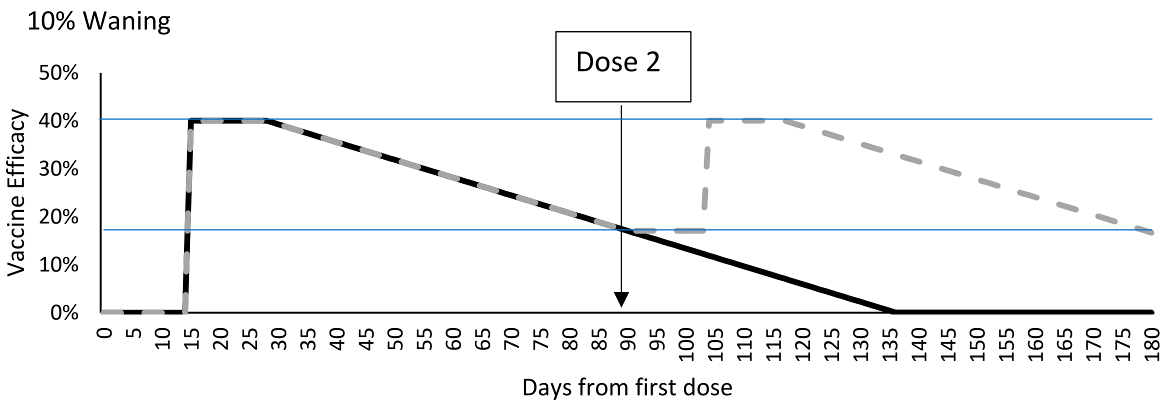

Dose 1, Sept through Nov			Dose 2, Dec through Jan		
September	October	November	December	January	February
October	November	December	January	February	March
November	December	January	February	March	April

5. **Simulation Scenarios**

Agents were randomly chosen for application of prior immunity and vaccination. The basic study design was to model the typical one dose annual influenza vaccination pattern [8] relative to a range of seasonal influenza case peaks (Figure 1). In all models, individuals age <65 years were vaccinated beginning September 1 over a mean of 45 days with a standard deviation of 14 days (Table S2). For agents ≥65, individuals in the base model received a single influenza vaccine in a similar distribution during the same time period, beginning September 1, as those <65 years. In the comparison model, individuals 65 years and up received two influenza vaccine doses. In this model, the first dose was administered with the same distribution as the single dose model and the second dose was administered 90 days after the first dose.

Table S2. Simulation scenarios. Vaccine efficacy started at 40% two weeks after each dose and was sustained at this level for two weeks before waning began. For two dose primary comparison, all who received the first dose received the second dose.

	**First vaccine dose**		**Second vaccine dose**	
	**Date**	**Distribution**	**Date**	**Distribution**
Base simulation, Single vaccination normally distributed and completed by December	September 1 Start date	Normal over ~90 days, μ = 45 and σ = 14*	None Administered	
Comparison simulation, Two vaccine doses, second dose 90 days after the first dose	September 1 Start date	Normal over ~90 days, μ = 45 and σ = 14*	December 1 Start date	90 days after first dose

*The second dose was exactly 90 days after the first. A mean of 1.7% of agents were vaccinated beyond the 90-day window (i.e., in December for their first dose) when using the normal distribution.

6. Supplemental Results

Table S3. Percentage reductions in cases and hospitalizations compared to base model of one influenza vaccine dose. Negative values represent an increase.

**Peak Month**	Two Doses		Decreased Second Dose Uptake		Decreased Second Dose VE		Decreased Waning of Both Doses		Oct 1 Dosing vs. Single Dose*	
By Age	Cases	Hospital	Cases	Hospital	Cases	Hospital	Cases	Hospital	Cases	Hospital
**December**										
Age 65-74	1.8%	2.9%	0.6%	0.6%	1.5%	2.6%	1.1%	0.7%	-0.9%	-0.3%
Age 75-84	1.3%	2.0%	0.2%	0.1%	0.9%	1.9%	0.8%	1.1%	-0.4%	0.2%
Age 85+	1.6%	1.4%	0.6%	0.9%	1.4%	1.2%	2.5%	2.5%	0.9%	0.0%
Total Cases ≥65	1.6%	2.2%	0.5%	0.5%	1.3%	2.0%	1.1%	0.0%	-0.6%	-0.1%
**January**										
Age 65-74	5.6%	6.0%	4.4%	4.4%	4.7%	5.3%	3.9%	4.6%	4.2%	4.7%
Age 75-84	4.2%	5.0%	3.8%	3.8%	2.7%	3.1%	2.8%	2.5%	2.9%	3.5%
Age 85+	3.9%	4.8%	2.9%	3.7%	1.9%	3.1%	2.5%	-0.3%	1.9%	1.5%
Total Cases ≥65	5.0%	5.3%	4.1%	4.0%	3.9%	4.0%	3.4%	2.6%	3.7%	3.4%
**February**										
Age 65-74	15.8%	15.0%	12.9%	13.0%	11.2%	11.8%	12.3%	9.8%	4.3%	4.8%
Age 75-84	12.5%	12.2%	10.6%	11.8%	7.7%	6.8%	9.5%	6.1%	3.9%	4.3%
Age 85+	10.7%	11.0%	8.7%	8.0%	6.0%	5.4%	6.5%	11.5%	3.1%	3.3%
Total Cases ≥65	14.4%	13.1%	11.9%	11.4%	9.8%	8.5%	11.0%	9.8%	4.1%	4.3%
**March**										
Age 65-74	20.4%	20.2%	13.0%	12.4%	13.8%	14.4%	16.7%	17.5%	1.4%	1.7%
Age 75-84	16.3%	14.5%	9.8%	10.0%	11.6%	11.0%	11.7%	11.5%	1.6%	1.2%
Age 85+	13.5%	14.4%	8.2%	9.2%	9.1%	9.5%	8.7%	8.9%	1.3%	1.3%
Total Cases ≥65	18.7%	16.8%	11.8%	10.8%	12.8%	12.1%	14.9%	13.3%	1.4%	1.4%

*Negative values represent no reduction, i.e., an increase.

7. References

1. Krauland MG, Galloway DD, Raviotta JM, Zimmerman RK, Roberts MS. Impact of Low Rates of Influenza on Next-Season Influenza Infections. *Am J Prev Med*. Apr 2022;62(4):503-510. doi:10.1016/j.amepre.2021.11.007

2. Kumar S, Grefenstette JJ, Galloway D, Albert SM, Burke DS. Policies to reduce influenza in the workplace: impact assessments using an agent-based model. *Am J Public Health*. Aug 2013;103(8):1406-11. doi:10.2105/AJPH.2013.301269

3. Kumar S, Piper K, Galloway DD, Hadler JL, Grefenstette JJ. Is population structure sufficient to generate area-level inequalities in influenza rates? An examination using agent-based models. *BMC Public Health*. Sep 23 2015;15:947. doi:10.1186/s12889-015-2284-2

4. Centers for Disease Control and Prevention. Influenza (Flu), General Population Vaccination Coverage. Accessed July 27, 2022, https://www.cdc.gov/flu/fluvaxview/coverage-1920estimates.htm#figure7

5. Centers for Disease Control and Prevention. Estimated Influenza Disease Burden, by Season. Accessed July 28, 2022, https://www.cdc.gov/flu/about/burden/past-seasons.html

6. Centers for Disease Control and Prevention. FLUVIEW Interactive, Laboratory-Confirmed Influenza Hospitalizations. Accessed July 28, 2022, https://gis.cdc.gov/GRASP/Fluview/FluHospRates.html

7. Czaja CA, Miller L, Alden N, et al. Age-Related Differences in Hospitalization Rates, Clinical Presentation, and Outcomes Among Older Adults Hospitalized With Influenza-U.S. Influenza Hospitalization Surveillance Network (FluSurv-NET). *Open Forum Infect Dis*. Jul 1 2019;6(7)doi:10.1093/ofid/ofz225

8. Centers for Disease Control and Prevention. Influenza Vaccination Coverage for Persons 6 Months and Older. Accessed July 27, 2022, https://www.cdc.gov/flu/fluvaxview/interactive-general-population.htm

9. Centers for Disease Control and Prevention. Past Seasons Vaccine Effectiveness Estimates. Accessed July 28, 2022, https://www.cdc.gov/flu/vaccines-work/past-seasons-estimates.html

10. Ferdinands JM, Fry AM, Reynolds S, et al. Intraseason waning of influenza vaccine protection: Evidence from the US Influenza Vaccine Effectiveness Network, 2011-12 through 2014-15. *Clin Infect Dis*. Mar 1 2017;64(5):544-550. doi:10.1093/cid/ciw816

11. Ferdinands JM, Gaglani M, Martin ET, et al. Waning Vaccine Effectiveness Against Influenza-Associated Hospitalizations Among Adults, 2015-2016 to 2018-2019, United States Hospitalized Adult Influenza Vaccine Effectiveness Network. *Clin Infect Dis*. Aug 16 2021;73(4):726-729. doi:10.1093/cid/ciab045
